# Foreign

**Published:** 1840-09-12

**Authors:** 


### FOREIGN.

### PHILLIPS' LECTURE ON THE PRINCIPLES AND PRACTICE OF SURGERY.—NO. VI.

DISEASES OF JOINTS *generally.—Those of particular Tissues.—The* SYNOVIAL MEMBRANE. —*The* ARTICULAR CARTILAGES.—SCROFULOUS DISEASE.—DISEASES OF PARTICULAR JOINTS.—*The* HIP *and the* KNEE—*their Characters, their Diagnosis, and their Treatment.*

#### SYNOVIAL INFLAMMATION.

This affection may extend from adjoining parts, or begin in the membrane itself; but we confine ourselves here to the latter. It seldom attacks young children, is less rare towards puberty, is frequent in adults; this is the reverse of what happens with respect to some other of the diseases of the joints. Where it is a consequence of some constitutional cause, such as gout, rheumatism, syphilis, &c., it is usually not very severe. It may succeed to physical injuries, but the most frequent source of the disease is cold. It may leave the joint with its functions impaired or may totally destroy it. For the most part it is a chronic or slow inflammation, which, while it impairs, does not altogether destroy the functions of the joint. Pain is at first usually referred particularly to one spot; it increases for a week or ten days, and even then may not be very great; sometimes, however, it is very distressing. In a day or two after the pain begins, swelling may be apparent; this swelling at first arises from a preternatural collection of fluid in the joint. When the inflammation has existed for some time, the fluctuation is less perceptible, because the membrane becomes thickened. The form of the swelling is peculiar; it results from distension; the ligaments at certain points resist its progress, and give it a somewhat lobulated appearance. After synovial inflammation has subsided, the fluid is absorbed, and in some instances the joint regains its natural figure and mobility; in others, the stiffness and swelling remain, and the patient is then very liable to a recurrence of the disease. In some cases the disease is more acute; the swelling is then almost coeval with pain, the skin is red, the pain severe, and greatly aggravated by motion at the part; with symptomatic fever. In a few days, if left to itself, the disease may assume a chronic form, or subside altogether.

*Treatment.*—When the disease is complicated with rheumatism, opium, combined with ipecacuanha, or other diaphoretics, colchicum and mercury, may be preferred. Where many joints are affected, colchicum wine, in doses of fifteen to thirty minims, three times a day, I prefer. Where only one or two joints suffer, calomel and opium, in such doses as to affect the system, may do best. Where syphilis has to do with it, a well regulated course of mercury will probably cause it to disappear. In most cases, however, our principal reliance must be on local remedies. In the acute form of the disease, leeches and general bleeding may be required. If the swelling be sudden, and the pain be great, warm fomentations and poultices may relieve; otherwise, cold lotions do better. Chronic inflammation does not so easily subside; the joint must be kept perfectly quiet; blood must be abstracted locally by cupping or leeches, (the former is the surer and preferable mode;) it may be necessary to repeat it two or three times; in the intervals, cold lotion must be kept to the part. When the inflammation has subsided, one or a succession of large blisters may be used. If the joint be much distended, it may be punctured. The puncture, if it be small, will give only present relief, for the joint will quickly fill again. If suppuration has taken place, a free opening into it will often be attended with the best effects. The most prudent method is first to puncture with a needle, and ascertain the nature of the fluid; if actual pus, the lancet may be used afterwards. When inflammation is in a great measure relieved, rubefacients are often useful. Issues and setons are useful in chronic cases. No other active remedies seem to be of much use; but much may be done by negative treatment, especially by absolute rest. Stiffness and thickening may be much relieved by slight moxas, by passing a red hot iron very lightly and quickly over the surface, or by friction after the plan of Grosvenor; but this remedy should always be used with caution, as it has sometimes occasioned the return of inflammation. Warm douches are also useful, as well as the vapour bath, but all these methods require time and patience. It is often very difficult, without, or even with a previous history, to determine whether synovial inflammation, or "ulceration of cartilages," has been the primary affection; but this is of less importance, because, whatever the origin, when it has proceeded far, the treatment is the same; and generally, when suppuration has taken place, removal of the limb alone promises much success.

THICKENING OF THE SYNOVIAL MEMBRANE.

As to *thickening* of the synovial membrane, in which that tissue loses its natural organization, and becomes converted into a thick pulpy substance, of a light brown, or sometimes of a reddish brown colour, the disease, says Sir B. Brodie, generally takes place not long after puberty. In the origin of this disease there is a slight stiffness and tumefaction, without pain or much inconvenience. These symptoms gradually increase. In the greater number of cases, the joint at last scarcely admits of the smallest motion. The form of the swelling bears a certain resemblance to that of synovial inflammation, but it is less regular. The swelling is soft, elastic, and gives the sensation of a fluid. There is little or no pain till abscesses form, and the cartilages ulcerate; the abscesses heal more readily, and discharge less pus, than in the latter disease. At this period hectic is developed, and, unless the limb be removed, the patient gradually sinks. The progress of this disease is variable; generally two years elapse before it arrives at its last stage. The *diagnosis* is seldom difficult. The gradual progress of the enlargement, and stiffness of the joint, without pain, and the soft elastic swelling without fluctuation, in the majority of cases enable us to distinguish it readily from all the other morbid affections to which the joints are liable, except those of chronic inflammation of the synovial membrane, with which it may be confounded.

*Treatment.*—We must not be sanguine that a remedy will be discovered for this affection; very many have been tried and failed. By means of rest and cold lotions, the progress of the disease may be somewhat checked. If there be much pain from ulceration of cartilages, some benefit may be derived from warm fomentations or poultices; but no method seems to be capable of more than checking, somewhat, the progress, and relieving symptoms. In every case, towards the termination, there is ulceration of the cartilages, formation of abscesses, and consequent general disturbance, which render amputation necessary as a means of saving life.

Many illustrative cases of these several varieties of synovial disease may be found in his very able work. "A man was admitted into St. George's Hospital, with swelling of the knee, accompanied by pain, rigidity, and fluctuation. These symptoms yielded to the use of liniments and blisters. Two months after he died of fever. Upon examination, the synovial membrane was found distended, so that it was pushed out to about the extent of an inch and a half beyond its ordinary limits. Throughout, except where it covered the cartilages, it was of a dark colour, resulting from inflammation, and was as much injected as the conjunctiva in ophthalmia; at some points coagulable lymph lined the capsule. A young man felt a painful tumefaction in one knee, which he attributed to cold. Different remedies were employed; another disease of the leg required amputation, and the knee was examined. The bones, cartilages, and ligaments were in a natural state; the synovial membrane was the eighth of an inch thick, and converted into a cartilaginous structure, and was strongly adherent to the cellular tissue around. A girl, of nine, fell on the hip; she suffered little inconvenience, walked out during the day, and danced at night, but felt a rigor which obliged her to return home and go to bed. The next day she complained a good deal of the hip, and of the knee of the same side; fever was developed, which increased, until she became delirious, and in a week she died. Twenty-four hours after death her body was examined; the viscera were healthy, but the hip-joint contained half an ounce of dark pus, and the synovial membrane, at the point where it is reflected upon the head of the femur, was ulcerated to the extent of a shilling. A man having suffered a contusion of the shoulder, it was followed by tumefaction and severe pain at that joint. A nervous fever, which attacked him after the accident, destroyed life in a few days. Upon examination, half an ounce of pus was found in the shoulder joint. The synovial membrane was inflamed and ulcerated to the extent of a sixpence, at the point where it is reflected upon the neck of the humerus. In all these cases we see that this membrane alone was affected, presenting the different degrees of inflammation that we have admitted. Most commonly, however, the disease extends farther, invading other tissues of the joint, as we shall see hereafter.

*Ulceration of Cartilages.*—Can articular cartilages ulcerate? So great an authority as Brodie, we find, is of opinion that they can, at any period of life, but that the most frequent instances are found between the period of puberty and thirty-five; that it usually affects a single joint, but may attack two or three; that it forms the great majority of those cases of caries of the hip-joint which occur in adult persons.

It is, and has long been, customary to describe inflammatory action, and the many changes it brings about, as affecting the synovial membrane, covering the articular cartilages. Nesbitt and Hunter first spoke of this covering, but never demonstrated it. Bichat and his disciples only admitted it analogically, and few persons have contradicted them. Gordon, many years ago, said that it was a mere anatomical refinement, but his opinion rested on assertion only. John Bell and Dorsey asserted that diseases never began in it; Cruveilhier and Magendie have reiterated it. Certainly the scalpel demonstrates that it terminates at the circumference of the articular facettes. The transparent pellicle, which can be detached by separating, slowly, a slice of a diarthrodial surface, is, I apprehend, a portion of the cartilage itself, and has no relation with the adjacent synovial membrane. Exposed in a living animal, it may remain an indefinite time in contact with the air, may be touched and irritated, without ever becoming injected, reddened, tumefied, or in the least painful, whilst the proper synovial membrane is intensely inflamed. The general opinion is, that if the tissue of articular heads be injected, it is spongy, easy to see that instead of expanding and diverging in the cartilage, the arteries and veins form arches in the osseous tissue itself; that cartilages are formed of perpendicular filaments, or superposed plates, if they be not homogeneous, containing neither vessels, nerves, nor cellular tissue, being in all things similar to the enamel of the teeth, from which, according to Cruveilheir and Larrey, they only differ in cohesion and hardness; that their appearance is so fixed that nothing can change it; that in disease there is no mention of redness; that if they disappear during disease, it is by "erosion," solution, or molecular absorption; that, however much may be destroyed, what remains is unchanged; in fact, that it is physically altered, but not diseased. Morand found, in examining Madame Supiot, in whom all the bones were softened, that the cartilages of the hip and the knee were perfectly healthy. This opinion Sir B. Brodie has long combatted; and, to a certain extent, his views have lately received a kind of confirmation, which was previously wanting; Mr. Liston having, it is believed, succeeded in injecting cartilage. He very kindly allowed me to inspect his very beautiful specimens, and I am not prepared to say that the opinion of their being injected is incorrect. That the injection passed along a canal in the inflamed cartilage I cannot doubt, but the injection did not seem to me to branch out or radiate from this canal through the cartilage; it passed along to the surface, and there was fairly extravasated between the cartilage and a layer of lymph, and partly, perhaps, penetrated that lymph. It is true that there are other tissues in which vascularity cannot be demonstrated in the state of health, though it can when they are inflamed; but in those cases vascularity is then very decided, which is not the case with cartilage.

Sir B. Brodie maintains that articular cartilages may be primarily inflamed and ulcerated; that the ulceration may and frequently does begin on its articular surface, and that the inflammation may extend to the other tissues of the joint; that the cartilages may here and there be changed into a fibrous mass, that they may be ulcerated at many points, and that the articulation may be filled with fetid pus; and that this affection is altogether different from ordinary inflammation of the synovial membrane, and the spongy extremity of bones. To this view of the case it is objected, that the so-called ulcerations were mechanical destruction, or a breaking down by caries; certainly, in most of the cases, there was a softened or carious or tubercular bone below the ulcer; the cartilage not being tumid or vascular.

It is said that this inflammation and ulceration may be seen at all ages, but that it is most frequently seen in young persons; most commonly in the hip and shoulder joint.

The only symptoms of this disease met with for some time, says Brodie, are pain; and if the articulations of the lower extremities be affected, pain and a slight degree of lameness in the lower limb. The pain at first is trifling and only occasional, afterwards severe and constant. It resembles a good deal the pain of rheumatism, since it has often no certain seat, but is referred to different parts of the limb. As the disease advances, the pain becomes exceedingly severe, particularly at night, when the patient is continually aroused from sleep by painful starting of the limb. As the pain increases in intensity it is more confined in its situation. Wherever the pain is situated, it is aggravated by the motion of the joint; but it is aggravated in a still greater degree by pressure of the articular surfaces one against another. This disease may be confounded with inflammation of the synovial membrane, the scrofulous affection having its origin in the bones; a painful nervous affection occurring in hysterical females; and certain affections of the sciatic nerve.

The principal *diagnostic* mark is the pain which is experienced at the beginning, unattended by swelling, and which is invariably increased by pressing the articular surfaces one against another. The pain is referred to the point which is the actual seat of the disease. Whatever joint is affected, the formation of abscesses is always attended with an aggravation of the symptoms; but the degree in which the general system is disturbed when suppuration is established, depends on various circumstances—the age, the power of the patient, size of the joint, and its situation. In its progress the disease is generally tedious.

The *prognosis* is always unfavourable, for at its commencement the disease is frequently mistaken, and it rapidly brings about structural change in the constituents of the joint.

*Treatment.*—It is, of course, important to attend to the patient's general health. Undoubtedly there is no medicine of which it can be said that it exercises a specific influence over the disease; but Brodie cannot doubt that a course of sarsaparilla properly prepared and administered in full doses, is often productive of the greatest benefit. When the cartilages of a joint are ulcerated, it may well be supposed that the motion of their surfaces on each other must be favourable to the progress of ulceration. "I have known some cases in which rest alone was sufficient to produce a cure." I have employed caustic issues, and seen them employed in a great number of cases, and have found them usually to be productive of singular benefit when the cartilages are in a state of ulceration. Blisters and setons seem to act nearly in the same manner as caustic issues. Local and general bloodletting is in an early stage of the disease productive of advantage, and in the same stage the warm bath will be found serviceable. Friction is invariably injurious.

*Scrofulous disease.*—In scrofulous disease of joints, the cancellous structure of the bones is the part primarily affected, "in consequence of which, ulceration takes place in the cartilages covering the articular surfaces." The cartilages being ulcerated, the subsequent progress of the disease is in many respects the same as when the ulceration of the cartilage takes place in the first instance. I apprehend many of these cases in which the texture of the bones is softened, and yellow cheesy substance was deposited in their cancelli, were cases of tubercular deposition, such as I have already described. "This disease is frequent in children; rare after thirty: it may affect several joints together or in succession: it is rarely met with except in persons who have marks of a scrofulous diathesis. It is not unlikely to be confounded with ulceration of cartilages. Before the disease has extended beyond the cancellated structure, or any swelling is apparent, pain is experienced; generally, however, it is not severe; after a time, parts external to the joint sympathise; tissues become infiltrated, and the joint appears swollen; the swelling is puffy and elastic. If no suspicion of disease existed before, it is always awoke by the swelling. It increases, but not uniformly, and especially after exercise. As the cartilages continue to ulcerate, pain is aggravated, but is not severe until abscess has formed: the abscess bursts, or is opened, and thin curdy pus discharged; it becomes gradually thicker, and very closely resembles the cheesy matter of scrofulous glands; other points give way, and continue fistulous; it may remain so for months without occasioning much disturbance: in some cases hectic comes on, and unless the limb be amputated the patient sinks; at other times the sinuses close, the œdema subsides, and the patient ultimately recovers with or without anchylosis.

*Treatment.*—In treating this condition it must be borne in mind that the system is at fault, and therefore general is almost as necessary as local treatment. I cannot say that the abstraction of blood from the part is never useful, but certainly it is seldom necessary. If there be an accidental supervention of inflammation, leeches and cold lotions may be employed with advantage. It rarely happens that good is obtained from blisters or liniments, or counter-irritation. I much doubt whether setons and issues are ever useful, except there be great pain and spasm. There is, however, one rule respecting local treatment, which is applicable to all cases, and which can never safely be disregarded. The diseased joint should be kept perfectly quiet, because motion is likely to promote ulceration, and hasten the formation of abscess. During the formation of abscess, fomentations and poultices should be used to relieve pain. When, after the formation of several abscesses, the disposition to suppurate lessens, anchylosis, as a curative process, is about to commence; circular pressure around the limb will then be useful. As to constitutional treatment, a residence on the sea-coast is desirable; the diet should be plain, but nourishing. It appears to me preparations of steel are more useful than any other medicines; they must be continued, with slight intermissions, for a long time, until their exciting action may render it prudent to suspend them. In some cases, the liquor potassæ, with bitters, is useful; in others, iodine and its compounds will be found to act beneficially. When the organization of the joint is destroyed, and the health is failing, recourse must be had to amputation.

In the preceding description of articular inflammation affecting severally the tissues of a joint, I have adhered as closely as was compatible with the necessary condensation to the text of Sir B. Brodie, except as will have been seen, questions have arisen, as in ulceration of cartilages.

There are two joints where these affections are often seen, and where a more detailed consideration is necessary—the hip and the knee.

### COXALGIA OR MORBUS COXARIUS, is a complex affection of the hip joint, the nature of which is not unlike the so-called *white swelling* of other joints. Hippocrates speaks of it, (Aphor. sect. 6, Aph. 59 and 60;) and he even knew the actual cautery used for its cure. Asclepiades, the Bythnian, also speaks of two cases in which dislocation had occurred; Galen believed it to be owing to a relaxation of the ligamentum teres. The Arabs seem to have had more definite ideas of the disease. Albucasis points out a cause, which was indicated afterwards by Petit, in the first work of any importance on the subject: the Arab says that it sometimes happens that mucus accumulates in the hip-joint and causes luxation. We recognise it by the increased length of the diseased limb, and the void which is observed at the displaced point. Petit is the first person who well described the disease. It particularly affects children from three to fourteen, yet it may be seen at an earlier period: Camper has often seen it in children of eighteen months; Morgagni at a still earlier period; and Richter gives cases in which the disease was congenital. But it may be developed during adult life. Ficker met with it in a person of thirty-eight; Albers in one of forty-five; Paletta and Kraack in men of fifty.

The disease usually begins by a pain in the hip; more or less acute if it succeed to a blow over the trochanter; dull and deep seated when not excited by accident; sometimes only felt at intervals, at others having an erratic, rheumatic character. It increases, however, becomes more fixed, is felt above or below, though sometimes at the level of the articulation, sometimes in the groin; it is increased by pressure and motion of the limb, and it is then usually very acute. Sometimes it is felt at the knee or along the whole limb. The pain at the knee or the ankle is often so severe as to mask the hip disease. The sign which Boyer pointed out "that pressure on the knee does not increase the pain," is, I believe, a theoretical idea, for instances to the contrary are often seen. The absence of tumefaction at the knee is not always a distinguishing sign. The pain is often accompanied by contraction of the flexor muscles, so as to oblige the patient to walk on his toes. There is usually developed about the same time a fulness of the superior part of the thigh. Still, although the patient is lame, there is no very decided change in the length of the limb; and it is not easy to state upon what this lameness depends: to a certain extent it may be owing to feebleness of the limb; to some extent to pain. If at this time the two limbs be measured, we shall probably find the diseased limb a trifle the longer.

Such are the three principal characters of this period of the disease—pain, elongation of the limb, and lameness. To these, a new series of symptoms succeed: the elongation is replaced by a shortening, varying in extent with the age of the subject, and to circumstances to which we shall presently advert. The shortening is accompanied by all the symptoms of luxation upwards and outwards; that is, the knee and foot are rotated inwards; the great trochanter being directed upwards and forwards. It may occur suddenly: thus Desault has seen shortening occur to the extent of two inchess in a single night. In many cases the great trochanter is drawn upwards by the glutæi muscles; then the shortening supervenes without any change in the direction of the limb. Again, in some cases there supervenes suddenly an elongation or increased elongation of the limb, sometimes assuming the appearance of luxation into the foramen ovale, by the contraction of the pectinæus and other muscles.

Inflammatory action goes on, and pus is formed in and around the articulation; it approaches the surface, and points. We recognise it by the more defined tumefaction, diminution of pain, and fluctuation. The most common situation of purulent collections, is the external part of the thigh; sometimes they are seen at the internal surface. When they are marked by inflammatory symptoms, they usually point in the buttocks. According to Dzondi, it is at this time the acute pain at the knee is felt. Whether we open these abscesses, or leave them alone to break, they pour out ill conditioned pus. The openings often close after a short time, and new ones are formed and become fistulous; the patient becomes emaciated, and sometimes sinks under the severity of the pain and the abundance of the suppuration, which produce hectic fever and death. I should mention that cases have occurred where abscess and death have supervened without any change in the length of the limb. In those cases Boyer believed that caries was limited to the circumference of the cotyloid cavity.

If we have opportunities of examining the bodies of those suffering from this disease at an early period of its existence, we shall find the soft parts external to the joint infiltrated with purulent serum; here and there it is collected in the form of abscess. Sometimes these collections are considerable before any external opening has been formed; the muscular tissue, through which this fluid is disseminated, is much changed, and the aponeurosis is perforated. If the abscesses have burst, we shall see fistulous canals extending to the joint, the original seat of irritation. The articular capsule is often partially destroyed; sometimes it is not perforated; and there is occasionally a communication established by the breaking down of a part of the circumference of the cotyloid cavity. The fatty matter, termed synovial glands, and found at the fundus of the cavity, sometimes becomes tumid, and expands to such an extent as nearly to fill the cavity. The ligamentum teres is sometimes strained and elongated, but more frequently completely destroyed; the same fate attends the periosteum covering the neck of the femur and the border of the acetabulum. The cartilages covering the head of the femur and the cavity of the acetabulum are thickened and softened, according to some; strained and destroyed, according to others. However, we frequently find bones denuded, and, consecutively, caries is often developed in the contact with pus. Sometimes caries affects the fundus of the cavity, and the purulent matter may find its way into the pelvis; may even perforate the organs contained there. Sir A. Cooper refers to two cases in which it had found its way into the rectum. Often the head of the femur is the principal seat of alteration; at other times, in the midst of much disease of the cavity, it is little affected.

The relations of parts seem to be changed by the destruction of parts; the femur, shortened by the alteration of the head, sinks deeper into the cavity, also hollowed by caries, so that the occasional shortening may be thus explained. indeed, Larrey believes this to be the ordinary explanation of it, and that displacement is exceptional. However, we find the head of the bone in the iliac fossa with sufficient frequency to establish that as a not very unfrequent cause of shortening.

When the bone is displaced, the limb shrinks; the muscles are enfeebled or atrophied by want of use. Nelaton believes that this atrophy is accompanied by a decrease in the length of the femur; this atrophy, which does not confine itself to the femur, but to a certain extent affects the leg, must not therefore be forgotten in making out the cause of shortening. Petit attributed this dislocation to an accumulation of synovial fluid, which distends the capsule, causes pain, and relaxes the ligaments, so as to render them unable to resist the tendency to displacement upon motion. Sabatier, Desault, and Boyer denied this dropsy, which they had never seen, but which others have. A case is described by Lesauvage, of Caen, in the Archives Gen. 2de serie, tome 9, which directly supports Petit's opinion: a case is also mentioned by Brodie. Those who do not deny the existence of dropsy of the joint, yet say, while the accumulation is going on there should be lengthening up to the moment of displacement, when the limb would be drawn up by muscular action. There can, however, be no question, I apprehend, that lengthening of the limb does occasionally happen, but I believe Sir B. Brodie's explanation will account for many cases of apparent lengthening. He believes that in all cases the lengthening is only apparent, not real, and that it is caused by obliquity of the pelvis. The body resting on the healthy side, the pelvis of that side becomes elevated, and the other side proportionably depressed. The inclination of the pelvis is necessarily accompanied by a lateral curvature of the spine, and then one shoulder is higher than the other. All these symptoms may disappear at the end of a few weeks, if the horizontal position be preserved, unless the deformity have existed long, and the patient be young. From this direction of the hip, an apparent lengthening of the limb is the necessary result. He believes that the shortening which is sometimes presented at an early period, has nothing real in it; but putting aside both shortening and lengthening, he believes the disease to be an inflammation of the synovial tissue of the joint, or a scrofulous affection of the bone itself; that its most frequent cause is primary ulceration of the cartilages, progressive destruction of the osseous surfaces, and, as a consequence, a true shortening. Boyer admits as probable, that at the commencement of the disease the cartilage which lines the cotyloid cavity, that which covers the head of the femur, the ligamentum teres, and the so-called synovial gland, are tumefied; that this tumefaction, by destroying the proportion which ought to exist between the diameter and depth of the cavity and the head of the femur, produces that elongation which he thinks is almost always observed at this period. He mentions the case of a person who died from the consequences of luxation, consequent upon contusion; he discovered that the displacement resulted from the cartilaginous surfaces; that this tumefaction had almost effaced the cotyloid cavity, and elongated the head of the femur. The etiology of Petit having been abandoned, many persons have inclined to the opinion that luxation is commonly produced by inflammatory tumefaction of the diarthrodial cartilages, the synovial apparatus, and the ligamentum teres. Among the supporters of this opinion are Schwenke, Gorter, Vermandios, Van Swieten, Camper, Callisen, Plenck, Dehaen, Portal, and Boyer. If we find sarcomatous masses in the cotyloid cavity, certainly the displacement is easily explained. Yet these sarcomatous masses, and even the tumefaction of cartilages and the synovial apparatus, are not constantly met with, even after the dislocation has been effected. I recollect a case in which the head of the femur was entirely carious, and smaller than usual; it had escaped from the acetabulum and rested on the descending ramus of the ischium; there was no trace of round liniment, but the cotyloid cavity was very little changed. Observations like that induced Rust to seek other explanations; he sought to prove that the disease begins in the head of the femur; that it commences with inflammation of the medullary membrane, with a tendency to ulceration; that it ends by degenerating into a deep-seated central caries, and that this caries extends from the centre to the circumference; that at a later period the head of the femur, enormously swelled, obliges the great trochanter to direct itself downwards and outwards; that an elongation is thus produced, which may extend to four inches. At other times, on the contrary, the muscles draw the head upwards, as it is expelled, producing a shortening which may cease at intervals, and is never so complete as that which supervenes as a consequence of consecutive luxation. He adds, that if post-mortem inspections do not always support this view, it is because in those cases inflammation and caries of the cotyloid cavity were the consequences of disease previously existing in the pelvis.

A careful examination of a large number of specimens preserved in museums has convinced me that the head of the femur is more frequently diseased than the cotyloid cavity: the head of the femur may unquestionably be attacked with central caries, and this caries may extend from the centre to the circumference. Still, I am far from thinking that the theory of Rust applies to all cases of hip disease. Caries of the head of the femur, or of the cotyloid cavity only belong to one variety of the disease. How does the theory of Rust explain the dislocation, when the head of the femur, instead of being larger, is smaller than natural? atrophied. Berard saw the case of a grandson of Condorcet, in which the head of the femur on the diseased side was more than a third larger than the healthy one; the acetabulum had enlarged to contain it, and the articular cartilages were healthy. Dzondi, admitting the correctness of Boyer's reasoning as to lengthening, which he believed to be constant in the first period of the disease, maintains that the disease is always caused by rheumatic irritation, affecting the external surface of the capsule as well as the fibrous tissues connected with it, as well as the periosteum which surrounds the cavity and the superior part of the femur. Fricke maintains that two diseases have been confounded under one denomination: the one coxalgia, caused by relaxation of the muscles without disease of the joint; the other, a true inflammation of the articulation. In the first, elongation is constant; in the second, there is acute pain; and to ease that pain, there is vigorous muscular action, by which the head is firmly pressed upon the cavity so as to create a shortening.

In all these explanations, two things must not be confounded, the facts and the explanations. Brodie, Bichat, and Rust's observations are incontestible, though different—but are their explanations equally incontestable? My own opinion is, that the lesion is complex, and, therefore, that the appearances upon examination are not identical. Now, with respect to the pathognomonic symptom, lengthening, it is certain that since attention has been directed to the inclination of the pelvis, it is rarely met with; we are, therefore, bound to admit that former observers overlooked the circumstance. It is clear, then, that the elongation of the first period may be simulated, by the depression of the pelvis on the diseased side; or being real, the cause remains to be determined; that in the second period the symptom may be real, and a consequence of luxation into the foramen ovale; that the shortening in the first period may be simulated by the elevation of the pelvis on that side, or it may be real, and be owing to muscular contraction, which draws up the head of the bone, pushed out of the cavity, by an accumulation of synovia, the tumefaction of the soft parts within the articulation, or other cause; that in the second period it is owing to the destruction of the articular surfaces; to a displacement into the iliac fossa, or, according to Dzondi, to a change in the direction of the neck of the femur. Many or all of these causes may concur to produce this affect; for instance, we may find, in the same case, atrophy of the limb, ulceration of the articular surfaces, and a vicious inclination of the pelvis.

In making an examination for the purpose of determining whether there be any change in length, there are three positions in which the patient may be placed; first, lying on the back; then we put our thumbs on the anterior and superior iliac spines, mark their relation to an ideal horizontal line, and draw similar lines at the knees and the ankles. Sanson proceeds in the following way: from the upper part of the sternum he drops a line which represents the axis of the body; from one iliac spine to the other he draws a tape, which crosses the first; if the two spines be on a level, these tapes will fall perpendicular the one to the other; if, on the contrary, one hip is higher than the other, the angles will be unequal, and the most obtuse will be on the side where the hip is depressed. Brodie's method is, I think, hardly so exact as Sanson's: he extends the tape from the iliac spine to the superior margin of the patella of one side and the other. In case of elevation or depression of the pelvis, despite apparent change of length, the measure will be the same on both sides. Dzondi made the patient sit upon a chair, so that the lower part of the back shall touch the seat; the legs are carried forwards parallel to each other; the transverse line of the pelvis making a right angle with the thigh. At first he compares the two knees, then raising at the same time both feet, and flexing them strongly, the legs being in a right line with the thighs, both heels are then compared, and if they pass each other, the case is clear. Whatever may be the advantages of examining the patient sitting and lying, the erect position is unquestionably useful; it enables us to judge of the inclination of the spine and the pelvis, and to observe the character of the buttocks, a matter of first rate importance; for, certainly, one of the most common signs of ulceration of the cartilages is a remarkable flattening of the corresponding buttock, which becomes flabby, and looks for this reason larger than that of the opposite side, though, usually, in reality, it is not so. This appearance seems to be owing to a falling away in the firmness and vigour of the glutæi muscles; it is upon this sign that Brodie mainly relies, in determining whether the condition be one of ulceration of the cartilages, or inflammation and tumefaction. But I do not think that this symptom can be relied on, to distinguish between these two affections. Again, Brodie relies upon the character of the pain, which, according to him, is less acute in inflammation of the hip-joint, and is not accompanied by the excruciating pain which accompanies ulceration of the cartilages; but of the decided value of this symptom I doubt. Still, I think the pain ought to be attentively considered; it is often only evident upon pressure, yet the absence of pain is not sufficient to affirm that there exists no alteration of the hip, for in some cases it is wanting.

It is important to consider what are the affections which we may confound with spontaneous luxations of the femur. I do not think it necessary to do more than caution you against confounding this disease with fracture of the neck of the femur, of the crest of the ilium, or luxation of the femur, though doubtful cases will occur; nor with congenital luxation; in that, lameness is coeval with the attempt to walk, and there is no pain. It is not, however, easy to distinguish this affection from certain chronic diseases. The pain in the knee may induce error, even when it is associated with pain in the hip, and change in the length of the limb; it may often induce mistake; it may be taken for disease of the coxo-femoral articulation, when it only affects the sacro-iliac symphysis. Again, it is not always easy to distinguish between coxalgia and rheumatism of the hip; indeed, the distinctions are not defined; the erratic character of the pain, which, before affecting the hip, may have visited other parts, is an important sign; and may not rheumatism impress disease upon the synovial, fibrous, and osseous tissue of the hip? Again, sciatica is usually sufficiently marked by the course of the pain. Chronic suppuration developed in the vicinity of the hip, in consequence of caries or necrosis of the great trochanter, or of the crest of the ilium, should be distinguished from those which are a consequence of coxalgia; by the smaller quantity of pain attending motion of the thigh, and the absence of remission during repose; suppuration of psoas, presenting at the external part of the thigh, by the symptoms of psoas abscess, and by the absence of pain when the head of the femur is pressed down. Still difficulties will arise, such as when a chronic abscess opens into the acetabulum, either by perforating the fundus of the cavity, or in any other way.

There is nothing fixed or certain about the progress of coxalgia; it is sometimes very rapid, passing through all its stages in a few weeks; sometimes it may drag on through months, or even years. The first period extends from the beginning of the disease to the displacement of the head, and is usually as much shorter as the disease has been excited by external violence, as it is more painful, and as the patient is more vigorous and plethoric. In children, it is usually rapid; possibly because of the comparative shallowness of the cavity; but where they are scrofulous, it may be prolonged for many months. It is, during this period, that we may hope to arrest the progress of the disease; we may see then the elongation, simulated or real, and the pain disappear, and motion of the limb becoming practicable; yet there usually remains a certain rotation of the limb outwards.

The examination of a limb, made long after the cure, does not ordinarily show any lesion; sometimes the diarthrodial cartilage is replaced by a complete eburnation of the osseous surfaces. During life, this eburnation is proved by a shock, experienced during the movement of the limb by the patient, and even perceptible to a bystander.

If medical assistance have been obtained too late; if by the carelessness of the patient, or the rapidity of the disease, it have been impotent, and displacement has occurred, the affection may proceed no farther; a false joint is formed, and the patient cured; presenting, as the case may be, a lengthening or shortening of the limb. This termination is not, however, the most common; oftener the pain persists, abscesses are formed, and fistulæ established, the patient sinking under the disease. This duration of the period is very variable; it often extends to a year before death; sometimes even then the fistulæ may close, and the limb become anchylosed; but, if this result is probable, care should be taken about the position of the limb. It is, therefore, evident that, in a case of this kind, the result must be very doubtful; it is also very uncertain what course a displacement may take.

If we examine the hip after a cure by anchylosis, we find the femur united to a point of the iliac fossa, or the cotyloid cavity; sometimes it forms a right angle with the trunk; sometimes it is parallel with the axis of the body, and the union is affected as in other cases of anchylosis.

After the cure by pseudo-arthrosis, or false joint, we find, on the place where the femur rests, a depression; at the same time the head of the femur is diminished in size, and is flattened, so as to have contact with the ilium by a larger surface. The new joint is supported by muscular fibres, which take a fibrous appearance; the whole limb is atrophied, as a consequence of long inaction.

A point of great importance in the *prognosis* of this disease, as a means of determining whether any mode of reduction should be employed, is to ascertain what is the state of the cotyloid cavity. The generally received opinion is, that it becomes effaced, filled up, either by the tumefaction of the soft parts it contains, or by the production of exostosis. Boyer thought that the cavity abandoned by the head of the femur is more deformed by the pressure which the bone exercises on it from without inwards than by any other cause, but that the cavity is usually preserved.

There is much difference of opinion as to the causes of the disease. Some authors, with Petit, regarding it as the result of external causes —a fall upon the trochanter; others denying this cause in any case, and believing that this disease is always caused by constitutional means. I think there can be no doubt that constitutional causes exercise greater influence upon the development of the disease than those of an occasional nature; at least, they favour the action of the accidental cause; it is, however, true, that a fall upon the great trochanter, the knee, or even the foot, the leg being in extension, frequently excites all the symptoms of the disease we are considering. Usually, however, no sufficient cause can be assigned for the existence of the disease. Some persons believe a scrofulous disposition to be the cause; others, rheumatic irritation; this Dzondi strenuously maintains. Larrey regards the disease as scrofulous in the earlier periods of life— rheumatismal in adult life. There are other debilitating causes, to which some men have given weight as occasional agents, masturbation, venereal excesses, arthritic diathesis, syphilis. Again, certain eruptive affections would seem to excite the disease; measles and small-pox; it sometimes follows bad fevers, parturition, and other causes.

*Treatment.*—This disease not being always identical in its nature or its progress, it is evident that its treatment cannot be identical; that it must be varied, not only with the periods of the disease, but with the nature of the cause, the constitution of the patient, and the intensity of the symptoms. I believe nothing to be of such paramount importance as absolute rest, prolonged through the treatment; and after the cure, or we may run considerable risk of relapse; but essential as this is to the success of the treatment, its attainment is not always easy: either we have to contend with the indocility of a child, or with the feelings of friends, who will not make up their minds to such a long confinement, for what they think a comparatively unimportant disease. Again, if the limb be secured effectually, the ennui of a child is often distressing. In consequence of this, many modes have been proposed for the purpose of obtaining the same result: by fixing the patient on a mattress, and when it is necessary to move at all, moving the entire trunk by means of a rope hanging from the bed head, the limb itself being rolled with junks, but I think the long splint is most effectual. When we have reason to expect a constitutional origin for the disease, the constitution should be modified by appropriate means. Bearing that in mind, the acute local symptoms which are often seen, should be energetically treated by antiphlogistics: they ought to be employed with a vigour proportioned to the intensity of those symptoms. The disease being set going by violence, the patient vigorous, the pains very acute, blood may be taken from the arm, and leeches or cupping glasses repeatedly applied to the part: at the same time the diet should be low. Baths have been recommended very strongly by Dzondi, but there is a serious objection inseparable from their use—motion of the limb. In fact, at an early stage of the disease, he holds that baths, hot flannels, a small blister just below the trochanter major, and tartar emetic, combined with opium, will cure nine cases out of ten. I apprehend, however, that the cases to which he alludes are the rheumatic ones. When inflammatory action is lessened, if the pain still persists, though blunted, revulsives should be employed around the joint; they may be applied at first if the disease be chronic. Among these agents we generally use tartar emetic ointment, croton oil, blisters, setons, moxas, caustics, and the red-hot iron. Boyer employed a succession of blisters, placed upon the anterior, superior, and external sides of the thigh. These are used so long as the pain is present. He was accustomed to apply, in succession, from three to a dozen; sometimes, at first, they will be very beneficial, and then suddenly will seem to increase the pain of the articulation; they should then be suspended, and antiphlogistics should be substituted for them until the new irritation subsides. Some surgeons prefer to a succession of blisters a less effective plan, a single one, upon which they keep up suppuration by means of savine ointment; some prefer a seton external to the aponeurosis of the thigh; this often lessens the pain in the knee, which is occasionally very distressing; this seton should be large, and placed over the tensor vaginæ femoris muscle. Cauteries are also applied around the joint, but though more powerful in their effect than blisters, they cannot be compared to moxas; those should vary in size with the age of the patient. When the eschar is detached, it may be dressed with some stimulant to keep up suppuration. As soon as one is about to cicatrise we make a second, a third, and so on until the desired result is obtained. Professor Rust holds that none of these agents can be compared with iron, heated to whiteness; he does not, however, use it in acute cases until after blood-letting. He heats the iron or irons (for he employs as many irons as he requires lines) to whiteness; the cauterising extremity is three inches long by nine lines broad, and in form like a reversed prism. If the patient be young, three rays are enough; if older and vigorous, five; these rays converge from above downwards, and are placed an inch distant from each other. The first passes through the centre of the buttock for six inches, and in the direction of the sciatic nerve; the second is not so long, it follows the depression which the trochanter major leaves behind it; the third may pass over the trochanter itself. They should remain in contact with the integument some seconds whenever we wish an eschar of a certain extent. Rust, who admits a constant elongation of the thigh, states that he has observed often after the employment of the cauteries, that the limb resumed its natural length. There can be no question of the great efficacy of this treatment in a large number of cases. Still, as it is more painful, and the patient's fears are alarmed by it, it may be a question whether we should use it before the failure of moxas; and these even should not be used until milder means are tried. Certain it is, however, that transcurrent cauterisation and moxas are the means to be employed in the last period of the disease; the effects must be maintained by rest as absolute as can be obtained, the limb being extended. When abscesses are formed and opened, the cure is rare, especially where the abscesses are large; still it is not impossible. It is much more unfrequently that such abscesses disappear without opening, or are followed by cure. Again, it is to moxas that we owe such cures, but then they must be applied in much greater numbers than in the first period. Larrey has applied with success as many as twenty in a period of between fifteen and sixteen months. We may also use with success very large blisters, such as have been recently recommended by Velpeau. If, however, the abscesses present an acute character, antiphlogistic means must be used, and it may be necessary to open them to lessen the irritation. If the abscess seems to be caused by caries, if counter-irritants do not repress it, an opening must be made, but as to the time and mode of doing so, much difference of opinion exists. Some persons who are strongly impressed with the bad consequences produced by purulent collections, recommend an opening to be made as soon as the formation can with certainty be detected; most men, however, wait until the quantity of pus collected is very considerable, raising the skin and producing pain. As much inconvenience would attend one course as the other; if opened very early we give counter-irritants no chance to excite the absorbents; if left to collect in great quantity, the muscles are damaged, the integument is thinned, and the chances of cure remote, whether it be left to open itself, or it be done artificially. We usually open them by a simple puncture, either with a trocar or a bistoury; in the latter case with or without a valvular opening; the opening is closed by means of adhesive straps, the puncture will commonly have to be repeated; at last it remains fistulous, and the patient sinks under the profuse suppuration and accompanying hectic. The hectic is attributed by some persons to the contact of air with the interior of the abscess; by others it is maintained that it occurs more quickly if we largely open them by means of the potential cautery and the bistoury. Rust believes that whether the opening be large or small the same quantity of air is admitted. To avoid these effects, he recommends that before opening an abscess, we should strongly irritate the skin which covers it, by passing over it, two or three times, a red hot iron; and when the tension and pain caused by the burn are passed, we incise one of the eschars through its whole length, for the purpose of evacuating the fluid. In this way. he seeks to produce an inflammatory condition, analogous to that which nature sets up before an abscess is opened spontaneously, and which favours the tendency of the parietes to coalesce when the pus has escaped, and produces in them a state predisposing to adhesion. When the abscess is very large, and the patient very feeble, he passes the trocar, heated to redness, through the tumour, carries a seton through the openings, and leaves it there thirty-six hours. Larrey follows the method of Petit, of Lyons, opening the abscess by a small knife heated to redness, and afterwards applying the cupping glass. Whatever method is adopted, the result is often fatal: occasionally, the suppuration lessens, the fistulæ are healed, and the case does well. Whatever may be the period of the disease when the cure is obtained, or the means by which it has been brought about, great precautions are necessary to prevent relapse; the horizontal position should be long maintained, and when the patients get up they should move about on crutches; and much care must be devoted to the first attempts to rest the body on the diseased limb. Such are the means which may be employed in the treatment of coxalgia; these means may often succeed in the first period, rarely in the second; indeed, until of late years, the efforts of the surgeon were limited to obtain a cure by anchylosis, or, in a still more favourable case, to the formation of an artificial joint. Certainly, when caries is extensive, the most favourable termination that can be looked for is anchylosis, the limb being in extension; otherwise the limb will not only be useless, but inconvenient.

Dislocation, by these means, has until lately been deemed incurable, and the patient has been doomed to perpetual lameness. In 1835, Humbert, after maturely examining the condition of the limb, became convinced that the cotyloid cavity persisted, that its contraction was inconsiderable, that the new adhesions formed with the head of the femur might be destroyed; he proposed that attempts should be made to reduce the luxation: those attempts were made and were successful. In six cases, after graduated extension, maintained during a time varying between five days and several months, he has procured reduction; but when reduction was accomplished, no relaxation of the extension was permitted for many days, indeed, not until the parts were supposed to be accustomed to their restored condition, and then only very gradually. This reduction, however, does not appear to bring the limb to its proper length on all occasions; disease of the spine or deformity of the pelvis may be the cause of this, and there may be atrophy of the limb, which will be incurable, but the patient may be able to walk without difficulty. In speaking of congenital luxation, this point was considered.

*Lond. Med. Gaz.*

---

*The Causes and Treatment of Strabismus, or Squinting. Summary of seventy-six Operations by Mr. Liston.*

Sir,—Since the publication of some remarks on the subject of division of the muscles of the orbit in a late number of your Journal, I have assisted at seventy-six operations of this nature, performed by M. Liston, the result of which may prove interesting to some of your readers. I am, Sir, your obedient servant,

W. R. Ancram.

London, July 14, 1840.

The causes of contraction of the muscles of the orbit appear in several instances to be similar to those which produce shortening of other muscles of the body, and induce deformity in the extremities, as in club-foot, contraction of the hamstring muscles, &c., being dependent on direct or indirect injury or irritation of the nervous system. Under this head may be placed twenty-one of the cases which have been operated on; three being produced by direct injury to the brain, thirteen by the convulsions to which children are liable in early life, and nine by the irritation of the diseases of early childhood, small-pox, measles, and scarlatina. Habit is another rather frequent cause; the eye has been affected by some disease; in the anxiety to avoid the light it has been turned inwards, and permanent strabismus has been the consequence; or a shade has been worn over the eye, the light has been admitted only at the inner angle, and to this point the eye has been constantly directed; ten patients date their deformity from this occurrence. Only four persons have presented themselves who suffered, from their attempts to imitate their squinting companions, by permanent strabismus. Violent exertion, as in hooping-cough, occasioned the disease in five patients; and the action of the muscles of the eye, in looking suddenly at some object, caused a permanent contraction in seven others. Four cases were occasioned by falls. The relative proportion of congenital strabismus is small. I have found only eight who say they were born with the deformity, and in four cases I think that was rather doubtful. From my inquiries into the causes of strabismus, I should say that a far greater number of cases was occasioned by spasmodic contraction of the internal rectus, than by paralysis of the external straight muscle. In a few cases I omitted to inquire the cause of the deformity.

The contraction of the recti muscles varies in different individuals, from the slightest cast, with perfect power of motion, to almost complete immobility and partial concealment of the cornea in the inner angle of the eye. The cornea is most generally drawn simply inwards; in some cases downwards as well as inwards; more rarely upwards and inwards; of course, by the combined action of two muscles. Two patients have also presented themselves where the eye was drawn directly outwards, evidently bp a contraction of the external rectus. Both those individuals have been prevented by circumstances from submitting to the division of the abductor as yet; but it is proposed to perform the operation in the course of a few weeks. In a third case the external rectus has been divided, with the same success that attends the more common operation; it presented no greater difficulties than the division of the internal muscle. A most general effect of these changes is diminished power of sight, and this diminution increases in the ratio of the withdrawal and fixation of the cornea from the axis of the vision.

The causes which produce strabismus being usually the diseases of early childhood, that is the age at which the affection most commonly comes on. In the greater number of cases the disease has begun previously to the third year, and after that time the liability to the affection appears to decrease with the age. After puberty it is exceedingly rare. Of the fifty-three cases I have examined, only one occurred at the age of fifteen, and one at twenty-five; in the latter case, the strabismus was occasioned by direct injury to the head. As far as I can judge, both sexes are equally liable to this deformity; forty females and thirty-six males having submitted to the operation. The disease does not appear to attack one eye in preference to the other, nearly an equal number of right and left eyes having been operated upon. One case has been treated where both eyes were affected. Age is no bar to the performance or success of the operation. Patients of all ages, from four to sixty-two, have been treated for this affection. A contracted internal rectus, of sixty years' standing, is the most confirmed case in which I have seen this operation performed; it succeeded perfectly. In very young children it would hardly be advisable to attempt the operation when the strabismus was only of few months' duration. The displacement of the eye might depend on some disease of the brain or intestinal canal, which, by attention to the general health, might be removed, and the squint cured without resort to the more violent remedy of division of the muscle.

With respect to the operation, the safest way to secure its complete performance, is to dissect, perfectly clean, the internal part of the ball of the eye, for the slightest fibre of the internal muscle that remains undivided will prevent eventual success. In some instances, after the internal rectus has been cut across, the patient possesses the power of turning the eye a little inwards and downwards; this arises from contraction of the inner border of the inferior rectus. Mr. Liston is in the habit of partially or completely dividing this muscle in all these cases, and this has been done in about ten instances. The only and best criterion of the completion of the operation, is the total inability of the patient to move the cornea towards the inner canthus, from the centre of the orbit. If this exist, there is little fear of any return of the distortion of the eye; and to effect this purpose, it will sometimes be absolutely necessary to divide the inner border of the inferior rectus. In four instances the operation has not been perfectly successful; these were all early cases, where, after the division of the internal muscle, the patient still possessed the power of moving the eye somewhat inwards, and before experience had shown the necessity of the division of the depressor. Three of these cases have submitted to a second operation; in all the inferior rectus has been divided, and the cure is perfect. The fourth, a boy of thirteen years old, is fearful to submit to a second attempt; the eye is drawn downwards as well as inwards, caused, most probably, by contraction of the inferior rectus.

To insure the effect of the operation, the scissors and forceps certainly appear the most effectual instruments; and either for the clear dissection of the inner side of the eyeball, or for division of part of the inferior muscle, they are much to be preferred. By using these, the operator will avoid the danger of doing too little or too much; he will, on the one hand, make certain of cutting all the tendinous fibres which bind the eye in its unnatural position, and he will run no chance of wounding the sclerotic coat and evacuating the humours of the eye, and thus effectually destroying the vision. Ineffectual attempts to divide the internal rectus are common-enough occurrences; and cases have presented themselves where it has been necessary to resort to a second operation. The accident of destroying the organ has also happened, and is not unlikely to occur in the hands of those who go rashly and unskilfully to work.

Although such severe injury is done to the coverings of the eye, it is a rare circumstance to see any inflammation follow the operation. Only five cases have presented any such occurrence; and in all it was so slight, that the application of a few leeches to the inner angle of the eye soon relieved all the unfavourable symptoms.

A slight ecchymosis occurs under the conjunctiva, and may continue for some weeks, even after that membrane is entirely healed. In two or three cases a small abscess under the conjunctiva has formed, always of very little importance; it has discharged itself in a few days, and produced no inconvenience. A little caruncle, or large-sized granulation, sometimes springs from the conjunctiva, near the inner canthus; this may be snipped off if of large size, or its growth subdued by the application of sulphate of copper.

The restoration of the perfection of vision is one of the greatest recommendations of this beautiful little operation, and this effect has been most striking in several instances. In one very complete contraction of the internal rectus the eye seemed to have been perfectly disused, and incapable of estimating the distance of objects. This patient, when he shut the perfect eye, presented many of the phenomena of a person restored to sight by the operation for congenital cataract; and it was most interesting to remark the daily improvement in his sight. As the two eyes begin to correspond, both in acuteness of vision as well as uniform motion, some diplopia is occasionally complained of.

The first operations which were performed by Mr. Liston, took place in the early part of May. I have had an opportunity of examining lately some of these cases, and I find that the eyes remain perfectly straight. There appears no reason, therefore, to expect that the other cases will not succeed; and this enables me to say, that out of seventy-four cases one only has been unsuccessful, and this in the person of the young boy above alluded to.—*Lancet.*

*Ulceration of the Lung, originating in Caries of the Sternum.*—A full, powerful man, but of a decidedly strumous diathesis, was affected with circumscribed inflammation over the sternum, and oppression in the chest, about three months since; symptoms which he attributed to an accidental chill. The local appearances at this time were circumscribed œdematous, tumefaction; heat, pain, and redness, situated opposite to the mamma a little to the right of the mesial line. The patient complained of deep-seated pain and oppression beneath the externally affected part; there was cough, dyspnœa, and a slight expectoration. The general symptoms were febrile: quick, hard pulse; hot skin, thirst, white tongue, and constipated bowels. As the morbid process proceeded, the existence of pus became evident, and the swelling increased, with fluctuation, whilst the general symptoms assumed the hectic type. By the application of poultices the progress of the pus to the surface was facilitated, and after some days the abscess spontaneously broke, discharging ill-conditioned and offensive matter. The sternum was found to be carious; it was rough and excavated, and continued to discharge offensive pus as the morbid process increased. Notwithstanding active and judicious treatment, the disease continued to extend until the sternum was completely perforated and the lung exposed, which had doubtless contracted extensive adhesions by the opposing surfaces of the pleuræ during the increase of disease. The lung itself now became affected: a large ulcerated cavity was soon formed (most probably in the middle lobe of the right side) and air escaped easily by any respiratory effort. In this condition, with but slight aggravation of symptoms, he still continues, and at present he presents the following morbid signs, local and general. There is an ulcerating cavity at the point indicated, of about two or three inches in extent, through which air readily escapes in any powerful respiratory effort, but not during ordinary breathing; the margin of this cavity is red and tumid, and there is considerable tumefaction over a similar spot on the left side. The patient complains very little of pain, but the inflamed portions are extremely tender. There is occasional cough and expectoration, and the fever present is hectic: there is a permanently quick pulse, exacerbations of fever occurring in remittent paroxysms, occasional night sweats and thirst, whilst the strength fails rapidly; but there is no emaciation, as of late; the countenance has become puffy and œdematous, and of a leucophlegmatic aspect; and there is a decided tendency to general anasarcous effusion, as is evinced in the appearance and feel of the general surface.

Judging from the negative effect of treatment, the strumous diathesis, and the steady but decided advance of bad symptoms, the prognosis is unfavorable; whilst, on the contrary, the patient's youth, usual strength, and active habits, are in his favor.—*Ibid.*

